# From ESP-Permissive to ESP-Driven: Recontextualizing English in Indonesia’s Vocational Curriculum

**DOI:** 10.12688/f1000research.182978.1

**Published:** 2026-07-27

**Authors:** Putimasurai Putimasurai, Amirul Mukminin, Urip Sulistiyo

**Affiliations:** 1 Doctoral Program in Education, Graduate School, Universitas Jambi, Jambi, Indonesia; 2 Faculty of Teacher Training and Education, Universitas Jambi, Jambi, Indonesia; 3 Faculty of Teacher Training and Education, Universitas Jambi, Jambi, Indonesia

**Keywords:** English for Specific Purposes (ESP), vocational high schools (SMK), TVET, curriculum architecture, curriculum alignment, Indonesia

## Abstract

**Background:**

Technical and vocational education and training (TVET) increasingly expects English to support workplace communication. In Indonesian vocational high schools (Sekolah Menengah Kejuruan, SMK), English is formally positioned as vocationally relevant, but curriculum instruments provide limited guidance on occupational genres, needs analysis, and performance-based assessment. This study examined how English for Specific Purposes (ESP) is positioned and enacted in SMK Business and Management programs.

**Methods:**

This qualitative multiple-case study involved three purposively selected urban SMKs. Data comprised 19 curriculum documents, including national curriculum instruments and school-level planning documents, and semi-structured interviews with 21 stakeholders: six English teachers, nine students, three administrators, and three industry representatives. Documents were analyzed using qualitative content analysis, while interview transcripts were analyzed thematically. Findings were integrated through cross-case triangulation.

**Results:**

The analysis showed that current curriculum regulation creates space for contextualized English through school autonomy and vocational positioning. However, limited specification of programme-linked workplace genres, routinized needs-analysis procedures, and performance-oriented assessment shifted much of the recontextualisation work to teachers. Across the three cases, English teaching often remained closer to General English than sustained occupational genre work, although teachers, students, administrators, and industry representatives consistently valued more workplace-relevant English learning.

**Conclusions:**

The study identifies Indonesia’s current vocational English arrangement as ESP-permissive rather than ESP-driven. It proposes a tentative strategic framework for strengthening vertical alignment from policy to assessment and horizontal coordination among English teachers, vocational subjects, and industry partners. The framework is intended to guide curriculum refinement and future research on more coherent vocational English implementation.

## 1. Introduction

As economies navigate rapid technological change, workforce automation, and sustainability imperatives, vocational education is under intensifying pressure to align with evolving industry demands. Technical and vocational education and training (TVET) systems globally are being reimagined not only to support green and digital transitions but also to produce graduates who are agile communicators, capable of contributing in multilingual, cross-functional, and highly regulated environments ( UNESCO, 2015, 2022; Zhang et al., 2020). In this context, English is not simply a subject but a tool for operational fluency, enabling coordination across borders, access to manuals and standards, and compliance in documentation-heavy sectors ( Hyland, 2022; Roshid & Kankaanranta, 2023). Consequently, there is growing recognition that traditional General English (GE) instruction in vocational schools does not adequately prepare learners for these demands.

Pedagogical focus has therefore shifted toward English for Specific Purposes (ESP), which prioritizes communicative competence in discipline-specific settings ( Dudley-Evans & St John, 1998; Basturkmen, 2021; Hyland, 2022). From automotive maintenance to hospitality and health care, ESP provides a framework for integrating authentic workplace genres and practices into the classroom, helping to bridge the persistent divide between education and employment ( Bhatia, 1993; Arnó-Macià et al., 2020; Liu & Hu, 2021). ESP-aligned approaches that integrate needs analysis, genre pedagogy, and performance-based assessment are widely recommended because they orient teaching and assessment toward sector-specific communicative events and observable performance, rather than decontextualized language practice ( Arnó-Macià et al., 2020; Basturkmen, 2022; Dou et al., 2023). In curriculum terms, this implies that coherence depends not only on broad intentions but on alignment among learning outcomes, instructional tasks, and assessment evidence, a relationship that has been shown to shape the quality and comparability of classroom enactment ( Richards, 2013; Zhao et al., 2023).

In Indonesia, the current national curriculum regulation for vocational high schools ( *Sekolah Menengah Kejuruan/SMK*), including Kurikulum Merdeka with school operational designs ( *Kurikulum Operasional Satuan Pendidikan, KOSP*) and CEFR-benchmarked English learning outcomes, followed by the planned National Curriculum 2024, creates policy space for contextualised English in *SMK* ( BSKAP, 2024; Widodo, 2016; Syahida & Siminto, 2023).

However, historical experience with the 2013 Curriculum (K13) shows that the text types emphasised in schools (e.g., descriptive, narrative, recount) often sat tangential to vocational genres, blunting transfer to workplace communication ( Poedjiastutie & Oliver, 2017; Purwanti, 2018; Sari et al., 2024). Importantly, curriculum guides and standards do not operate only as statements of intent; they function as policy instruments that can either stabilise or loosen implementation depending on the specificity of the guidance and the assessment regime attached to it ( Beck, 2025). The present policy moment therefore offers an opportunity to articulate ESP more explicitly within programmes and sectors while avoiding past misalignments ( Albesher, 2023; Zhang et al., 2020; Syahida & Siminto, 2023).

Despite this opportunity, practice on the ground frequently remains GE-oriented, with limited codification of workplace genres, uneven use of needs analysis, and scarce moderated performance assessments ( Poedjiastutie & Oliver, 2017; Purwanti, 2018; Jiang et al., 2020; Pambudi & Kaliaskarova, 2023). These conditions produce variability across schools and raise concerns about fairness and labour-market signaling, as resource-rich SMKs more easily simulate authentic tasks and broker industry partnerships, while under-resourced schools tend to rely on generic materials ( Mukminin et al., 2019; Sari et al., 2024). In vocational systems, such variability is often amplified when competence-oriented expectations are not consistently translated into classroom assessment evidence and instructional routines, resulting in uneven realisation across sites ( Misbah et al., 2020). Strengthening ESP implementation in Indonesia is thus not only a matter of technical alignment with workplace needs but also a question of how curriculum regulation and system design shape who gets access to meaningful, work-relevant English learning opportunities.

To analyze this dynamic, the paper introduces a distinction between ESP-permissive and ESP-driven curriculum architectures. An ESP-permissive system acknowledges the relevance of ESP and may label English as a vocational subject, but it does not mandate explicit workplace genres, systematic needs analysis, or performance-based assessment. Implementation is largely left to individual teachers and local resources, which can result in fragmented practices. By contrast, an ESP-driven system embeds ESP principles structurally through clear genre specifications, routine and auditable needs-analysis mechanisms, lawful pipelines of authentic materials, and assessment systems that prioritize observable performance in workplace-like conditions. This distinction provides a conceptual lens for examining how Indonesia’s current national curriculum regulation creates permission but not pressure for ESP, and how this permissiveness contributes to uneven implementation across *SMK.*

Crucially, in this study the term ESP-permissive is treated as an analytically operational category rather than a rhetorical label. It is diagnosed when curriculum instruments authorise contextualisation but do not stabilise it through programme-linked genre targets, routinised needs-analysis procedures, and performance-oriented assessment requirements that make enactment auditable and comparable across sites. In policy terms, such outcomes are shaped by recontextualisation processes, namely how curriculum meanings are interpreted, translated, and enacted across levels of the system, often producing variation when instrument specificity is weak ( Hordern, 2021; Dickens, 2021; Piedra Moreno et al., 2025). This operationalisation guides the document analysis of the curriculum chain and is triangulated with stakeholder accounts to explain why vocational intent can coexist with persistent GE defaults in practice.

Existing research on ESP in Indonesia documents misalignments between curriculum frameworks and classroom practices, the dominance of GE-oriented textbooks, and resource-driven disparities in implementation ( Poedjiastutie & Oliver, 2017; Purwanti, 2018; Pambudi & Kaliaskarova, 2023; Sari et al., 2024). However, most studies focus either on classroom-level practices or on individual programmes. Less is known about how ESP is positioned within the national curriculum regulation and how different stakeholders, teachers, students, administrators, and industry partners, experience that positioning in everyday practice. There is, in other words, a need for a systems-level analysis that connects policy architecture, including its degree of permissiveness, with on-the-ground realities in vocational schools.

Responding to this gap, this qualitative case study examines how ESP is embedded in national and school-level curriculum documents and how key stakeholders perceive and enact vocational English in three purposively selected *SMK*s. By combining document analysis with multi-stakeholder perspectives, the study seeks to illuminate the structural and pedagogical features of an ESP-permissive system and to identify levers for moving toward a more coherent, ESP-driven model. Accordingly, the study addresses the following research questions:

1. How is ESP currently positioned within Indonesia’s vocational curriculum, and what barriers and enablers shape its realization in schools?
2. What strategic directions can guide a shift from this current positioning toward an ESP-driven implementation that is consistent across contexts and demonstrably improves students’ readiness for communication-intensive work?

## 2. Literature review

### 2.1 The global imperative for ESP in TVET

Technical and vocational education and training (TVET) systems worldwide are increasingly expected to prepare graduates not only for local labour markets but also for participation in regional and global value chains. In many sectors, English functions as a lingua franca for safety procedures, technical documentation, regulatory compliance, and client communication ( Hyland, 2022; Roshid & Kankaanranta, 2023). Against this backdrop, international policy documents frame TVET as a key vehicle for inclusive and sustainable development, emphasizing the need for curricula that are responsive to rapidly changing skill demands, digitalization, and green transitions ( UNESCO, 2015, 2022; Zhang et al., 2020).

Within this global agenda, English for Specific Purposes (ESP) has become central to discussions of vocational language education. Rather than treating English as a general school subject, ESP positions it as a tool for performing concrete tasks in particular occupational domains—such as writing maintenance logs in automotive engineering, drafting emails to vendors in business administration, or explaining procedures to clients in hospitality ( Bhatia, 1993; Arnó-Macià et al., 2020; Liu & Hu, 2021). TVET systems are therefore under pressure to move beyond General English (GE) and adopt ESP-oriented designs that make explicit links between language learning and workplace practice.

### 2.2 Core principles of effective ESP

The ESP literature converges on several core principles that characterize effective practice. First, systematic needs analysis is regarded as foundational. ESP courses are expected to be driven by an analysis of the communicative events, genres, and competencies required in the target situation, typically carried out through consultation with industry stakeholders, document analysis, and observation of workplace communication ( Hutchinson & Waters, 1987; Dudley-Evans & St John, 1998; Anthony, 2018). Without routine needs analysis, courses risk drifting back toward generic content that may not support learners’ actual occupational trajectories.

Second, ESP is closely associated with genre-based pedagogy. Professional communication is realized through recurrent text types—such as invoices, purchase orders, standard operating procedures (SOPs), reports, and minutes—that reflect the purposes, audiences, and conventions of specific discourse communities ( Bhatia, 1993; Hyland, 2006, 2022). Genre-based approaches aim to make these patterns visible and teachable, enabling learners not only to reproduce model texts but also to adapt them to new situations.

Third, the literature emphasizes authentic materials and performance-based assessment. Authentic texts and scenarios provide learners with exposure to the forms, lexis, and interactional norms characteristic of real workplaces, while performance-based assessment tasks require learners to demonstrate their ability to use English to complete meaningful work-like activities under specified conditions ( Arnó-Macià et al., 2020; Basturkmen, 2021, 2022; Dou et al., 2023). This stands in contrast to decontextualized tasks that test discrete language items without clear relevance to occupational communication.

Finally, recent work highlights the importance of multimodal and digital literacies. As workplaces increasingly rely on digital platforms, online documentation systems, and visual–verbal communication, ESP teaching must address not only written and spoken genres but also the integration of images, tables, interfaces, and digital tools ( Luzón & Pérez-Llantada, 2022; Hyland & Jiang, 2021). Together, these principles position ESP as a performance-oriented, needs-driven and genre-sensitive approach to vocational language education.

### 2.3 Operationalizing an ESP-permissive curriculum architecture

This study conceptualizes an ESP-permissive curriculum architecture as a policy–practice configuration in which vocational English is formally recognized as relevant, yet curriculum instruments do not sufficiently stabilize ESP enactment across schools. In such architectures, policy and curriculum documents may authorize contextualization, but they provide limited pressure and guidance for translating vocational intent into programme-linked classroom work. The concept is used here as an operational category rather than a rhetorical label and is examined through documentary evidence and stakeholder accounts.

ESP research consistently highlights three design commitments that support vocational relevance. First, systematic needs analysis is foundational for identifying target communicative events and occupational genres through stakeholder consultation and workplace discourse analysis ( Hutchinson & Waters, 1987; Dudley-Evans & St John, 1998; Anthony, 2018). Second, vocational ESP is inherently genre-oriented, because workplace communication is realized through recurring genres and registers that can be made teachable through explicit modelling and scaffolding ( Bhatia, 1993; Hyland, 2006, 2022). Third, ESP effectiveness depends on performance-oriented assessment, where learners demonstrate competence through work-like tasks under specified conditions and criteria, rather than through decontextualized language tests ( Arnó-Macià et al., 2020; Basturkmen, 2021, 2022; Dou et al., 2023). These commitments imply that curriculum coherence depends not only on broad proficiency aims but on whether curriculum instruments specify what counts as workplace-relevant performance and how it should be evidenced.

Building on these principles, this study operationalizes ESP-permissiveness through three forms of under-specification across the curriculum chain. The first is limited specification of programme-linked occupational genre targets. Curriculum outcomes and syllabi may signal vocational relevance, but they do not provide a clear set of priority genres and communicative events for particular vocational clusters, leaving task selection and sequencing largely to local interpretation. The second is the absence of routinized and auditable needs-analysis mechanisms. Where needs analysis is not embedded as a recurring procedure, vocational specificity depends on individual teacher initiative and local networks. The third is weak codification of performance-based assessment expectations, including limited requirements for assessable products, workplace-like performance conditions, and shared criteria. When these instruments are generic, classroom practice can default to General English materials and assessment formats even when policy discourse encourages contextualization.

This instrument-based conceptualization is particularly relevant in decentralized and resource-stratified contexts, where variability in industry access, authentic materials, and teacher capacity can widen differences in implementation across schools ( Mukminin et al., 2019; Pambudi & Kaliaskarova, 2023; Sari et al., 2024). In Indonesia, prior research has documented persistent misalignments between vocational intent and classroom practice and the continued dominance of GE-oriented materials under earlier curriculum arrangements ( Poedjiastutie & Oliver, 2017; Purwanti, 2018). The present study extends this work by examining how the national curriculum regulation and school-level curriculum documents encode vocational specificity and how stakeholders experience and negotiate these signals. Accordingly, the document analysis examines the extent to which regulation and guidance, learning outcomes, ATP, and Modul Ajar specify or under-specify genre targets, needs-analysis routines, and performance-oriented assessment, and triangulates these documentary patterns with multi-stakeholder interviews to explain how “permission without stabilizing instruments” shapes ESP enactment.

### 2.4 Implementation challenges in decentralized and resource-Constrained contexts

Translating ESP principles into classroom practice is challenging, especially in decentralized and resource-constrained education systems. Research in various contexts points to persistent misalignments between curriculum intentions and what happens in classrooms, limited access to authentic materials, and uneven teacher preparation for genre-based and sector-specific instruction ( Widodo, 2016; Jiang et al., 2020; Mukminin et al., 2019).

In Indonesia, these challenges are well documented. Studies of vocational English teaching under previous curriculum arrangements report that national syllabi and textbooks often foreground generic text types (e.g., descriptive, narrative, recount) that are only loosely connected to vocational communication needs ( Poedjiastutie & Oliver, 2017; Purwanti, 2018). Teachers frequently rely on centrally produced GE-oriented materials that do not specify workplace genres or industry-aligned outcomes, making it difficult to design tasks that mirror authentic communication in sectors such as office administration, accounting, or hospitality.

More recent studies continue to identify gaps in materials, assessment, and teacher support. Teachers report difficulty finding or developing texts that match actual job functions, especially when access to digital resources or industry networks is limited ( Rachmawati, 2025). Assessment practices tend to rely on traditional formats rather than task-based or performance-oriented assessment, despite evidence that such approaches can increase learner engagement and better reflect workplace demands ( Aminah et al., 2025; Nurisma, 2025; Pratama & Mukminatien, 2025).

Resource disparities further complicate ESP implementation. Schools in urban or relatively well-resourced settings may establish partnerships with industry, access online materials, and create project-based learning environments, whereas under-resourced schools often depend on outdated textbooks and disconnected syllabi ( Pambudi & Kaliaskarova, 2023; Sari et al., 2024). These differences raise equity concerns: students in different schools may receive markedly different opportunities to develop workplace-relevant English competence, even under the same national curriculum regulation.

Teacher capacity is another recurring issue. Teachers play a central role in implementing ESP in vocational schools; however, the professional preparation and systemic support required for this role remain limited. Many vocational English teachers report limited training in ESP, genre pedagogy, and assessment of performance, and they often work in isolation when trying to contextualize national guidelines for specific programmes ( Widodo, 2016; Mukminin et al., 2019; Asrifan & de Barros Cardoso, 2025). Without robust professional development, co-teaching arrangements with vocational teachers, or access to curated material banks, the burden of “translating” policy into ESP practice falls heavily on individual teachers.

### 2.5 Research gap: from local problems to systems-Level architecture

Taken together, the international and Indonesian literature offers a consistent diagnosis: ESP is widely regarded as essential for vocational education; its core principles are well defined; and common implementation problems—fragmentation, misalignment, and inequity—are well described. However, most existing studies focus on classroom-level practices, individual programmes, or particular schools. Less attention has been given to how national curriculum regulation and broader system design shape the conditions under which ESP can be implemented.

In particular, the literature has not systematically examined the difference between a curriculum that merely permits ESP and one that drives it. The former can be described as ESP-permissive: English may be labeled a vocational subject, and policy documents may encourage contextualization, yet there are no binding requirements regarding workplace genres, needs-analysis procedures, or performance-based assessment. Implementation is left largely to local interpretation and resourcing, which tends to produce variability across schools. The latter can be described as ESP-driven: ESP principles are embedded structurally in curriculum regulation, learning outcomes, materials pipelines, and assessment frameworks, creating both permission and pressure to align English teaching with workplace communication.

This distinction between ESP-permissive and ESP-driven architectures is not explicitly developed in existing studies of Indonesian vocational English. There is also limited research that combines detailed analysis of national and school-level documents with the perspectives of multiple stakeholders—teachers, students, administrators, and industry representatives—to understand how policy signals are interpreted and enacted in practice.

The present study addresses this gap by (a) examining how ESP is positioned within the national curriculum regulation and school-level documents for vocational English, (b) exploring how key stakeholders experience and negotiate that positioning, and (c) using the ESP-permissive/ESP-driven distinction as an analytical lens to propose strategic directions for a more coherent, system-level approach to vocational English in Indonesia.

## 3. Methodology

### 3.1 Research design

This study employed a qualitative multiple case study design to examine how English for Specific Purposes (ESP) is positioned and enacted within Indonesia’s vocational high schools (SMK). A case study approach was appropriate because the inquiry focused on a bounded phenomenon, namely the relationship between national curriculum regulation for SMK English, school level curriculum documents, and the interpretations and practices of key stakeholders in a small number of schools ( Yin, 2018). The intention was not statistical representativeness, but analytic generalisation through theoretically informed insights that may be transferable to comparable vocational contexts ( Patton, 2015). This qualitative orientation enables close examination of stakeholder meaning making and curriculum enactment in context ( Creswell, 2013; Merriam & Tisdell, 2015).

### 3.2 Study context

The study is situated in Indonesia’s *SMK* context, where curriculum regulation provides an official framework for English, while schools operationalize that framework through school level curriculum planning and lesson design. The analysis therefore connects curriculum instruments, including national regulation and learning outcomes guidance, with how vocational English is interpreted and enacted within schools and workplace related partnerships.

### 3.3 Sites and case selection

The study was conducted in three urban *SMK*s offering programs within the Business and Management cluster. Urban sites were purposively selected because demands for workplace relevant English, including written correspondence and service communication, are more visible in dense service sector settings, making policy to practice translation easier to examine. Business and Management was selected because it links to occupations such as office administration, accounting support, marketing, customer service, and sales, where English is frequently used for documentation, correspondence, and digital workplace communication.

To protect institutional identities while enabling cross case comparison, schools were anonymized using pseudonyms *SMK* A to *SMK* C. Table 1 summarizes the case sites and their programme focus.

**Table 1.** Site profile.

Site (pseudonym)	Location context	School status	Business and Management program focus	Industry engagement context	Rationale for inclusion
SMK A	Urban	State SMK	Office Management	Internship supervision and workplace communication exposure through partner organizations	Information rich case for examining vocational English in administrative communication contexts
SMK B	Urban	State SMK	Accounting	Internship related workplace documentation and correspondence expectations	Contrast case focused on documentation intensive work related genres
SMK C	Urban	State SMK	Marketing	Customer communication and promotional communication expectations during internship and school industry activities	Contrast case focused on service interaction and promotional discourse

Participants comprised 21 stakeholders across the three sites: six English teachers, nine students, three school administrators, and three industry representatives. Teachers were those teaching English in Business and Management programmes and involved in interpreting or preparing school curriculum documents. Administrators held curriculum or vocational coordination roles. Industry representatives were practitioners involved in internship supervision or school industry collaboration.

Recruitment was conducted through a transparent procedure designed to reduce gatekeeper bias and ensure voluntariness. Principals were approached through official letters and follow up visits, and schools that consented formed the case sites. English teachers and administrators were invited because of their direct involvement in vocational English. Student recruitment aimed to capture variation in achievement and gender. Teachers were asked to nominate a small pool of students reflecting diverse attainment profiles and gender representation, after which students were invited by the researcher and informed that participation was voluntary and had no academic consequences. Industry representatives were identified through existing partnership channels related to internships and programme collaboration and participated voluntarily. All participants were anonymized using codes: teachers T1 to T6, students S1 to S9, administrators A1 to A3, and industry representatives I1 to I3.

Inclusion criteria were defined for each participant group. Teachers were included if they taught English in Business and Management programmes and were involved in interpreting or preparing ATP or Modul Ajar. Administrators were included if they held curriculum or vocational coordination roles. Students were included if they were enrolled in Business and Management programmes and could reflect variation in achievement and gender, with priority given to those with internship exposure or readiness. Industry participants were included if they supervised internships or collaborated with the school on programme development. To mitigate gatekeeper bias in student recruitment, teachers were asked to nominate multiple candidates representing diverse profiles, after which invitations were issued directly by the researcher, and participation was voluntary. The final distribution of participants across stakeholder groups and case sites is summarized in Table 2.

**Table 2.** Participant distribution and role.

Participant group	Codes	Total n	Distribution across sites	Role in the case
English teachers	T1 to T6	6	Two teachers per site	Curriculum interpretation and classroom enactment of vocational English
Students	S1 to S9	9	Three students per site	Learner experience of vocational English and perceived workplace relevance
Administrators	A1 to A3	3	One administrator per site	School level curriculum coordination and implementation decisions
Industry representatives	I1 to I3	3	One partner per site	Workplace communication expectations and internship supervision perspectives

All student participants were 18 years old at the time of data collection and were therefore treated as adult participants. Although the study was conducted in vocational high school settings, no minor participants were involved. Student participation was voluntary, and each student provided informed consent before taking part in the interview.

### 3.4 Data collection instruments

**3.4.1 Curriculum documents**

Two sets of documents were collected. First, national curriculum instruments relevant to *SMK* English were examined, including the national curriculum regulation and associated guidance documents that shape school level planning. Second, school level documents used to operationalise English in the Business and Management cluster were collected from the case sites. These included the English learning outcomes guidelines (Capaian Pembelajaran), nine Alur Tujuan Pembelajaran (ATP), and eight teacher prepared Modul Ajar. These school documents were aligned with national guidance and were selected to represent planning across topics and learning sequences relevant to vocational communication, such as correspondence, oral interaction, and project work.

Documents were catalogued as a defined corpus with clear units of analysis. Units included outcome statements, learning objectives, task prompts, materials, and assessment sections. The document analysis protocol was developed from ESP principles and curriculum alignment constructs and focused on the degree to which documents specify vocational genres, traces of needs analysis or industry consultation, authenticity of tasks and materials, assessment orientation including performance based assessment, and linkages between English and vocational subjects (Bowen, 2009). The curriculum document corpus and its analytic focus are presented in Table 3.

**Table 3.** Curriculum document corpus and analytic focus.

Document type	Level	Quantity	Unit of analysis	Analytic focus
National curriculum regulation and associated guidance for SMK English	National	1	Clauses and sections relevant to English positioning, autonomy, assessment	Policy signals for contextualization, vocational specificity, assessment orientation
English learning outcomes guidelines *(Capaian Pembelajaran/CP)*	National	1	Outcome statements and descriptors	Vocational specificity and program differentiation
Learning Progressions ( *Alur Tujuan Pembelajaran/ATP*)	School level	9	Objectives, learning activities, assessment sections	Vocational genre targets, authenticity, needs analysis traces, performance orientation
Lesson Plans *(Modul Ajar)*	School level	8	Task prompts, materials, assessment artefacts and rubrics	Workplace simulations, observable products, criteria and rubrics, vocational purpose of digital tools

**3.4.2 Semi structured interviews**

Semi structured interviews were conducted with teachers, students, administrators, and industry representatives. Interviews typically lasted 35 to 60 minutes and were conducted primarily in *Bahasa Indonesia* to support participant comfort and depth. Separate interview guides were developed for each stakeholder group but shared common themes: the role and status of English in vocational programs, curriculum and workplace alignment, and experiences with ESP related practices, and perceived barriers and enablers under the current regulation. Interview guides followed a consistent structure that included introduction and consent, background information, perceptions of curriculum and learning experiences, challenges and recommendations, and closing reflections. All interviews were audio recorded with consent and transcribed verbatim. Where excerpts were presented in English, translations were produced conservatively and checked against the original transcript to preserve meaning.

**Figure 1.**
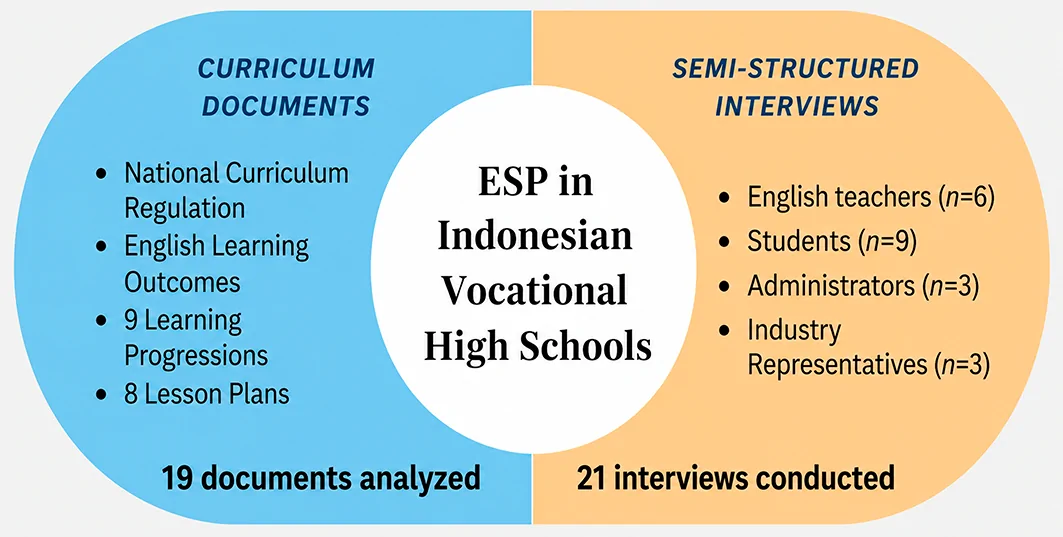
Data collection instruments.

For clarity and transparency, Fig. 1 summarizes the data collection instruments and the composition of both the document corpus and the interview sample.

### 3.5 Data collection and analysis process

Data collection proceeded in a staged sequence. Document collection and cataloguing were conducted first to identify curriculum signals and gaps that required probing. Interviews were then conducted to examine how these documentary signals were interpreted and enacted in practice and to capture stakeholder accounts of needs analysis, access to workplace genres, assessment practices, and cross subject coordination. A brief member feedback step followed, in which a summary of preliminary themes was shared with selected teachers and administrators to check resonance and clarify interpretation. The study was conducted within the dissertation fieldwork cycle, with core data collection and analysis carried out in 2025 and refinement and verification completed in 2026.

### 3.6 Data analysis

Analysis was conducted iteratively and comparatively. Curriculum documents were first analyzed to map policy signals and instrument specificity, interview transcripts were then analyzed to capture stakeholder enactment and constraints, and both strands were subsequently integrated through triangulation to support cross-case interpretation.

**3.6.1 Document analysis**

Curriculum documents were analyzed using a qualitative content analysis approach informed by ESP principles and curriculum alignment constructs. The analytic purpose was to examine how vocational English is framed across the curriculum chain and to identify the degree of specificity through which ESP is enabled or left to local interpretation. Analysis proceeded in two cycles.

In the first cycle, each document was read in full to establish familiarity with its structure and purpose and to define appropriate units of analysis. For national curriculum regulation and associated guidance, the units of analysis comprised clauses and sections related to the formal positioning of English, autonomy provisions, and assessment expectations. For the English learning outcomes guidelines, the units of analysis comprised outcome statements and descriptors. For *ATP* and *Modul Ajar*, the units of analysis included learning objectives, learning activity sequences, task prompts, instructional materials, and assessment sections, including any criteria or rubrics. A document log was maintained to record document identifiers, scope, and the segments coded.

Documents were then coded inductively for explicit and implicit references to six analytic domains derived from ESP scholarship and alignment literature and operationalized for the *SMK* context: vocational contextualization and programme differentiation; vocational genres and registers; needs analysis and industry consultation traceability; authenticity of materials and tasks; assessment orientation, including performance-based assessment; and horizontal linkages between English and vocational subjects.

In the second cycle, first-cycle codes were consolidated into higher-order categories to capture how curriculum instruments function across layers, from regulation and learning outcomes guidance to *ATP* and *Modul Ajar.* Categories were organized under two overarching dimensions. The first, policy positioning, captured how English is framed and what curricular space is provided for contextualization. The second, implementation specificity, captured the extent to which curriculum instruments stabilize ESP enactment by specifying genres, routinized needs-analysis procedures, and performance-oriented assessment expectations. Outputs from this phase included a structured mapping of supports and gaps across document layers, enabling comparison of where ESP is facilitated and where it remains under-specified.

Document analysis was conducted through manual coding supported by iterative memoing. Coding definitions and category revisions were documented in an audit trail to enhance transparency.

**3.6.2 Interview analysis**

Interview transcripts were analyzed using thematic analysis as a systematic, iterative procedure for identifying and refining patterns of meaning across stakeholder accounts. Analysis proceeded through six stages.

First, transcripts were read repeatedly for familiarization, accompanied by analytic memoing to capture salient issues, emerging contrasts, and potential explanatory mechanisms. Second, initial coding was conducted through line-by-line coding to generate descriptive codes grounded in participants’ accounts. Third, codes were collated into categories aligned with the study aims, including policy positioning of English, curriculum–workplace alignment, authenticity and access, needs analysis and industry dialogue, assessment practices, teacher capacity and workload, and equity and variability across schools.

Fourth, candidate themes were developed and reviewed for internal coherence and external distinctiveness. Theme refinement involved checking themes against the full dataset, accounting for divergent accounts, and ensuring that themes captured variation across stakeholder groups rather than reflecting a single perspective. Fifth, cross-stakeholder comparison examined convergences and divergences across teachers, students, administrators, and industry representatives. Sixth, cross-case comparison examined how themes replicated or varied across *SMK* A, *SMK* B, and *SMK* C.

Interview analysis was conducted through manual coding. Coding decisions, theme definitions, and revisions were documented through analytic memos and an audit trail to support dependability.

**3.6.3 Integration and cross-case synthesis**

Findings from document and interview analyses were integrated through triangulation to establish an auditable chain of evidence linking curriculum instruments to stakeholder experience and enactment. Integration proceeded in three analytic moves.

First, interview-derived themes were examined alongside documentary patterns to assess convergence, divergence, and compensatory practices, particularly where teachers described bridging gaps that were weakly specified in curriculum instruments. Second, documentary signals were interpreted in light of stakeholder accounts to clarify how policy space was taken up, constrained, or decontextualized at the school level. Third, integrated patterns were synthesized through the analytical distinction between ESP-permissive and ESP-driven curriculum architectures, focusing on whether curriculum instruments primarily permit contextualization or provide stabilizing mechanisms such as explicit genre targets, routinized needs-analysis procedures, and performance-oriented assessment expectations.

This integrative synthesis informed the presentation of findings by aligning documentary supports and gaps with stakeholder perspectives and cross-case contrasts to explain variability in ESP enactment across the three sites.

### 3.7 Trustworthiness

Trustworthiness was strengthened using credibility, transferability, dependability, and confirmability strategies ( Lincoln & Guba, 1985). Credibility was supported through triangulation across curriculum documents and stakeholder interviews, as well as member feedback, in which preliminary interpretations were shared with selected participants to confirm resonance and clarify possible misinterpretations. Transferability was supported through a clear description of the study context, case selection, participant composition, and document corpus. Dependability and confirmability were strengthened through an audit trail documenting corpus construction, coding iterations, theme refinement, and the linkage between claims and evidence, complemented by reflexive memoing to minimize interpretive bias ( Tracy, 2020).

### 3.8 Ethical considerations and consent:

This study was conducted after institutional and site-level research permissions had been obtained. The first author received an official research permission letter from the Doctoral Program in Education, Graduate School, Universitas Jambi, under letter number 106/UN.21.10.8/KM/2025, dated 19 May 2025. The permission letter was addressed to relevant school and industry sites involved in the study.

Formal ethical approval from an institutional review board was not obtained because the study was conducted as minimal-risk educational research involving curriculum document analysis and semi-structured interviews about participants’ educational and workplace-related experiences. The study did not involve clinical procedures, experimental treatment, sensitive personal data, or any intervention that could reasonably place participants at risk beyond ordinary educational research activities. Nevertheless, ethical principles were followed throughout the study, including voluntary participation, informed consent, confidentiality, anonymity, and the right to withdraw from the study at any stage without consequence.

Written informed consent was obtained from all participants before data collection. For the student group, all participants were 18 years old at the time of data collection and were therefore treated as adult participants. No minor participants were involved in the study. Students were informed about the purpose of the study, the nature of their involvement, the voluntary nature of participation, the confidentiality of their responses, and their right to withdraw at any time without penalty. Their participation was not connected to grading, assessment, or any academic consequence. Schools and participants were anonymized using pseudonyms and coded identifiers, including SMK A–SMK C for schools and T1–T6, S1–S9, A1–A3, and I1–I3 for participants. Interview recordings, transcripts, and related documents were stored securely with restricted access. The reporting of findings avoided disclosure of identifying institutional details, personal information, and proprietary workplace information.

## 4. Findings

This section reports findings from two complementary sources: (1) curriculum document analysis across the policy-to-classroom chain (national regulation and guidance, *CP, ATP*, and *Modul Ajar*), and (2) stakeholder interviews with teachers, students, administrators, and industry representatives. Findings are organised to address RQ1 by showing how ESP is positioned, what instruments enable or constrain enactment, and how these conditions are experienced in practice.

### 4.1 ESP in the national curriculum regulation and school documents

Across the document corpus, vocational intent is visible and policy space for contextualised English is created through time allocation, school autonomy, and project-based learning space. However, instrument specificity remains limited across layers. Three under-specified instruments recur: programme-linked workplace genre targets, routinised and auditable needs analysis mechanisms, and performance-based assessment requirements that specify assessable products, conditions, and criteria. The sections below trace this pattern across the curriculum chain.

**4.1.1 National curriculum regulation and associated guidance: policy space without enforceable ESP instruments**

The national curriculum regulation positions English as a vocational subject with a defined time allocation (108 intra-curricular hours plus 36 project hours). It also creates enabling conditions for contextualisation, including school autonomy through KOSP, formal space for interdisciplinary project-based learning ( *P5*), and extracurricular opportunities. These provisions constitute policy space for contextualised English.

At the same time, the regulation and associated guidance do not specify programme-linked priority workplace genres or communicative events, do not require a routinised and auditable needs analysis cycle, and do not frame performance-based assessment as a system-level requirement for vocational English. In effect, policy authorises contextualisation but does not stabilise ESP enactment through enforceable instruments.

**4.1.2 English learning outcomes: CEFR-Referenced benchmarks without vocational differentiation**

The *CP* specifies CEFR-referenced proficiency targets, including B1 as a general benchmark and B2 for an advanced track. These benchmarks provide a broad reference point for expected proficiency and signal that English should support communicative competence. The *CP* also contains limited references to English use in some vocational subject areas, suggesting potential cross-subject relevance.

However, *CP* outcomes for English remain largely homogenised across majors and do not specify programme-linked workplace genres and registers. Linkages between English outcomes and vocational subject outcomes are weakly articulated, and there is no systematic framework requiring needs analysis or documented industry consultation as an input to curriculum planning. Assessment expectations are also not framed in terms of workplace performance evidence. As a result, *CP* provides broad proficiency aims but limited direction for organising English around sector-specific communicative competence in Business and Management pathways.

**4.1.3 *ATP*: Vocational topics present, but weak genre modelling and performance specification**

Across the ATP documents, field- and workplace-related topics are present and occasional genre labels (e.g., business letters, emails) appear, which could support cross-subject connections. Nevertheless, most *ATP*s rely on generic text types without detailed genre models and include few clearly authentic or performance-oriented task sequences. Needs analysis is not documented, and performance conditions and criteria are rarely specified. Some *ATP*s also show phase mismatch, where higher-grade sequences repeat lower-phase task patterns, weakening progression. These features indicate vocational intent but limited stabilising instruments for consistent ESP enactment.

**4.1.4 *Modul Ajar*: Emergent vocational anchoring, but limited workplace simulation and assessable performance evidence**

The *Modul Ajar* show early attempts to anchor English learning in vocational domains. Genre-based instruction is visible, some real-world texts are introduced, and multimodal communication opportunities appear. In some modules, differentiated instructional pathways are evident.

However, alignment with workplace communication remains partial. Workplace simulations tend to be limited or fragmented rather than extended, workplace-like communication cycles. Performance-based assessment is not systematically specified as observable products under explicit conditions with shared criteria. Needs analysis is not documented in lesson planning artefacts, and digital tools are used without consistently stated vocational purposes. Interdisciplinary collaboration traces with vocational subject teachers are limited. Overall, these documents suggest movement toward vocational anchoring but insufficient instrument specificity to stabilise enactment across schools.

To make the ESP-permissive diagnosis auditable, the document analysis is summarised across curriculum layers, moving from national regulation and guidance to *CP*, *ATP*, and *Modul Ajar.* Table 4 presents the main ESP-enabling supports and the corresponding gaps in instrument specificity that shape how vocational English can be enacted in schools.

**Table 4.** ESP alignment across SMK english curriculum documents: supports and gaps.

Curriculum layer	ESP-enabling supports	ESP-critical gaps (instrument under-specification)	Implication for enactment
National curriculum regulation and associated guidance for *SMK* English	English positioned as a vocational subject; time allocation enables contextualized learning; autonomy through *KOSP*; formal space for *P5* and extracurricular learning	ESP not specified as an approach; no programme-linked priority workplace genres/communicative events; no routinized, auditable needs analysis requirement; performance-based assessment not specified as a system expectation	Policy creates space, but schools and teachers translate intent into ESP practices with limited scaffolding
English learning outcomes guidelines *(Capaian Pembelajaran/CP)*	CEFR-referenced targets (B1 general; B2 advanced track); limited references to English use in some vocational subjects	Outcomes homogenised across majors; workplace genres/registers not specified; weak linkage to vocational outcomes; no systematic needs analysis framework; assessment not articulated as workplace performance evidence	Broad proficiency aims exist, but vocational specificity and performance evidence are not stabilized
Learning Progressions ( *Alur Tujuan Pembelajaran/ATP*)	Field/workplace topics; occasional genre labels; potential for cross-subject connections	Generic text types without detailed genre models; limited authentic tasks and simulations; performance conditions/criteria rarely specified; needs analysis not documented; phase mismatch; teachers left to bridge gaps	Enactment depends on teacher initiative and local resources, contributing to uneven implementation
Lesson Plans *(Modul Ajar)*	Attempts to anchor lessons in vocational domains; genre-based instruction visible; some real-world texts and multimodal potential; differentiated pathways in some modules	Workplace simulations limited; performance-based assessment not systematically specified; needs analysis not documented; digital tools without clear vocational purpose; limited interdisciplinary collaboration	Vocational anchoring is emerging but not yet stabilized through assessable products, conditions, and shared criteria

These patterns indicate that the curriculum chain provides policy space for contextualization but offers limited instrument specificity for programme-linked workplace genres, routinized needs analysis, and performance-based assessment. This under-specification helps explain why recontextualisation work shifts to teachers and why enactment varies across schools.

### 4.2 Stakeholders’ perspectives On ESP integration in vocational english

Stakeholder accounts complement the document analysis by demonstrating how vocational English is interpreted and enacted in everyday practice. Across groups, vocational relevance is widely endorsed, but participants describe a system in which programme specificity, authentic materials, needs analysis, and assessment remain difficult to stabilise, resulting in teacher improvisation and uneven implementation.

**4.2.1 Reframing english: From general literacy to occupational relevance**

Stakeholders converged on the view that English should function as a vocational competence rather than only general literacy. Teachers emphasised the need for programme specificity and perceived a gap between policy framing and operational support: “the curriculum should not be universal but should be specific to English for each department” (Teacher 3). Students articulated programme-linked needs and requested workplace-oriented modules and simulations, such as billing emails and professional phrases for Accounting (Student 1) and weekly simulations for reporting or client communication (Student 2). Industry representatives likewise reported that graduates struggle with basic workplace documentation and professionalism: “they find it difficult to make office memos or minutes in English … the documents look unprofessional” (Industry 1). Collectively, these accounts indicate recognition of vocational intent but limited structural guidance for realizing it consistently.

**4.2.2 Authenticity and access to workplace genres**

Stakeholders repeatedly emphasized the need for authentic texts and scenarios. Teachers reported that textbooks are often too general and that they frequently search online for artefacts such as invoices and business emails (Teacher 2; Teacher 4). Students noted that many tasks remain school-based or fictional and requested workplace-relevant email tasks, such as billing or payment confirmation emails (Student 1). Industry participants confirmed that graduates often lack familiarity with workplace documents, for example quotations and vendor emails (Industry 2). At the same time, administrators and industry representatives noted legal constraints on sharing internal documents without agreements and anonymization. This creates a structural barrier: authenticity is desired, yet lawful access routes and anonymization protocols are not systematized, leaving authenticity dependent on local initiative.

**4.2.3 Industry dialogue and needs analysis**

Formal and routinized needs analysis for vocational English was largely absent. Teachers described industry engagement as informal and irregular, often limited to “casual talk during internship” (Teacher 2). Students reported limited experience with structured industry feedback for English. Industry partners indicated that they have internal training materials but cannot share them without formal agreements. Administrators suggested that standing industry boards, standardized needs analysis instruments, and shared genre banks could formalise dialogue. These accounts reinforce the document pattern that needs analysis remains under-specified as a system instrument.

**4.2.4 Projects and simulations in practice**

While policy provides space for projects, teachers described feasibility constraints linked to teaching load and time: “project-based tasks are good, but I rarely implement them fully … in the end, small tasks that can be finished in class” (Teacher 2). Students requested sustained simulations aligned to their majors, including service simulations and document chains (Student 8; Student 4). Industry partners expressed willingness to support co-designed tasks and co-assessment. This suggests that project space exists, but stabilizing instruments such as ready-to-use project briefs, sequencing exemplars, and shared rubrics are not yet institutionalized.

**4.2.5 From broad outcomes to concrete skills: The product-and-Conditions gap**

Teachers described CP outcomes as broad and similar to general senior high school expectations, and emphasized that vocational programs require explicit communicative products: “Accounting needs simple reports … Office needs correspondence … office document writing” (Teacher 2). Teachers also noted that they “rework the CP into ATP aligned with the major” (Teacher 4), indicating local translation work. Students and industry representatives similarly argued for outcomes framed as observable products under specified conditions and criteria (Industry 3; Administrator 2). This theme aligns with the document finding that performance-based assessment expectations are not stabilized in policy instruments.

**4.2.6 Teacher capacity and support**

Teachers described commitment to contextualizing English but highlighted the workload required to find authentic materials and redesign planning documents. They also reported limited formal structures for industry dialogue about English skills: “there has never been a formal discussion with industry about English skills … we depend on stories from students after internships” (Teacher 5). Administrators and industry representatives expressed interest in supporting ESP-focused capacity building, provided that templates and coordination mechanisms exist. These accounts clarify how ESP-permissive conditions shift the burden of recontextualisation to teachers.

**4.2.7 Autonomy, variability, and local curriculum practice**

*KOSP* autonomy was perceived as creating space for contextualisation. However, limited scaffolds to support autonomy were widely noted, and teachers described the *CP* as “too general” and requiring substantial effort to connect to workplace realities (Teacher 6). Students requested greater standardisation in professional templates and rubrics (Student 8). Industry representatives noted that retraining is often needed, including for emerging digital promotion roles (Industry 2; Industry 3). Administrators argued that autonomy would be more effective if paired with shared exemplars and secure authenticity pipelines, reducing variability while retaining contextual flexibility.

## 5. Discussions

The findings depict a vocational English system in which policy formally recognizes English as a vocational subject and creates space for contextualization, but leaves critical decisions about genres, needs analysis, and assessment largely to schools and individual teachers. This section interprets those patterns through ESP theory and curriculum recontextualisation and articulates levers for moving toward more coherent and equitable ESP enactment.

### 5.1 An ESP-Permissive system: Permission without stabilizing instruments

The combined document and interview evidence supports the interpretation that Indonesia’s current arrangement is best described as ESP-permissive. Policy creates space for contextualization through autonomy and time allocation, but does not stabilize enactment through enforceable instruments such as programme-linked workplace genre targets, routinized needs analysis, and performance-based assessment requirements. The reported necessity for teachers to “rework the *CP* into *ATP* aligned with the major” (Teacher 4) illustrates how translation work is displaced to local actors.

The ESP-permissive versus ESP-driven distinction advances a curriculum-architecture account of ESP policy by conceptualizing permissiveness as a function of policy instrument strength. Along a continuum from permissive to driven, stronger instruments (explicit genre targets, routinized needs analysis, performance-based assessment) reduce interpretive ambiguity and stabilize enactment, whereas weaker instruments increase reliance on local improvisation and amplify variability. In recontextualisation terms ( Bernstein, 2000), vocational intent is signaled, but the rules and mechanisms that compel translation into specific texts and assessable practices remain under-specified, helping to explain why GE defaults persist despite vocational aspirations.

### 5.2 Authenticity, boundary objects, and legal-Infrastructural constraints

Authenticity is central to vocational ESP because workplace genres function as boundary objects that connect classroom learning to professional communities ( Bhatia, 1993; Hyland, 2006, 2022). The findings show strong demand for authentic artefacts and scenarios, but also highlight legal and organizational constraints on sharing workplace documents. Without formalized agreements and anonymization protocols, authenticity remains dependent on teacher ingenuity and local goodwill. This positions authenticity not merely as a pedagogical preference but as a system design problem requiring institutional infrastructures for lawful sharing and curation.

### 5.3 Projects, performance, and the assessment gap

Policy instruments encourage project-based learning, yet enactment is constrained by time and workload, leading to smaller, manageable tasks rather than extended workplace simulations. ESP scholarship emphasizes that performance-based assessment is essential for aligning learning activities with workplace demands ( Basturkmen, 2021; Dou et al., 2023). The limited specification of performance conditions and criteria in planning artefacts helps explain why project potential remains under-realized. The system often collects evidence of learning that is weakly connected to the ability to perform workplace communication tasks.

### 5.4 Equity, autonomy, and system design

Autonomy under *KOSP* can support contextualization, but without shared exemplars, alignment protocols, and secure authenticity pipelines, it tends to generate variability rather than coherent localization. In resource-stratified contexts, this variability translates into equity risk, as schools with stronger networks and capacity are better positioned to simulate authentic tasks and broker industry input ( Mukminin et al., 2019; Pambudi & Kaliaskarova, 2023; Sari et al., 2024). An ESP-permissive architecture therefore not only shapes pedagogy but also distributes opportunities unevenly by shifting critical design work to local capacity.

### 5.5 Toward An ESP-Driven curriculum architecture: strategic framework

Building on the documented supports and gaps across the curriculum chain and the triangulated stakeholder accounts, this section articulates a strategic framework for moving from an ESP-permissive to an ESP-driven curriculum architecture. The framework does not propose a fixed national blueprint; rather, it identifies a set of alignment levers that, when activated collectively, can stabilise vocational ESP enactment and reduce reliance on local improvisation.

**Figure 2.**
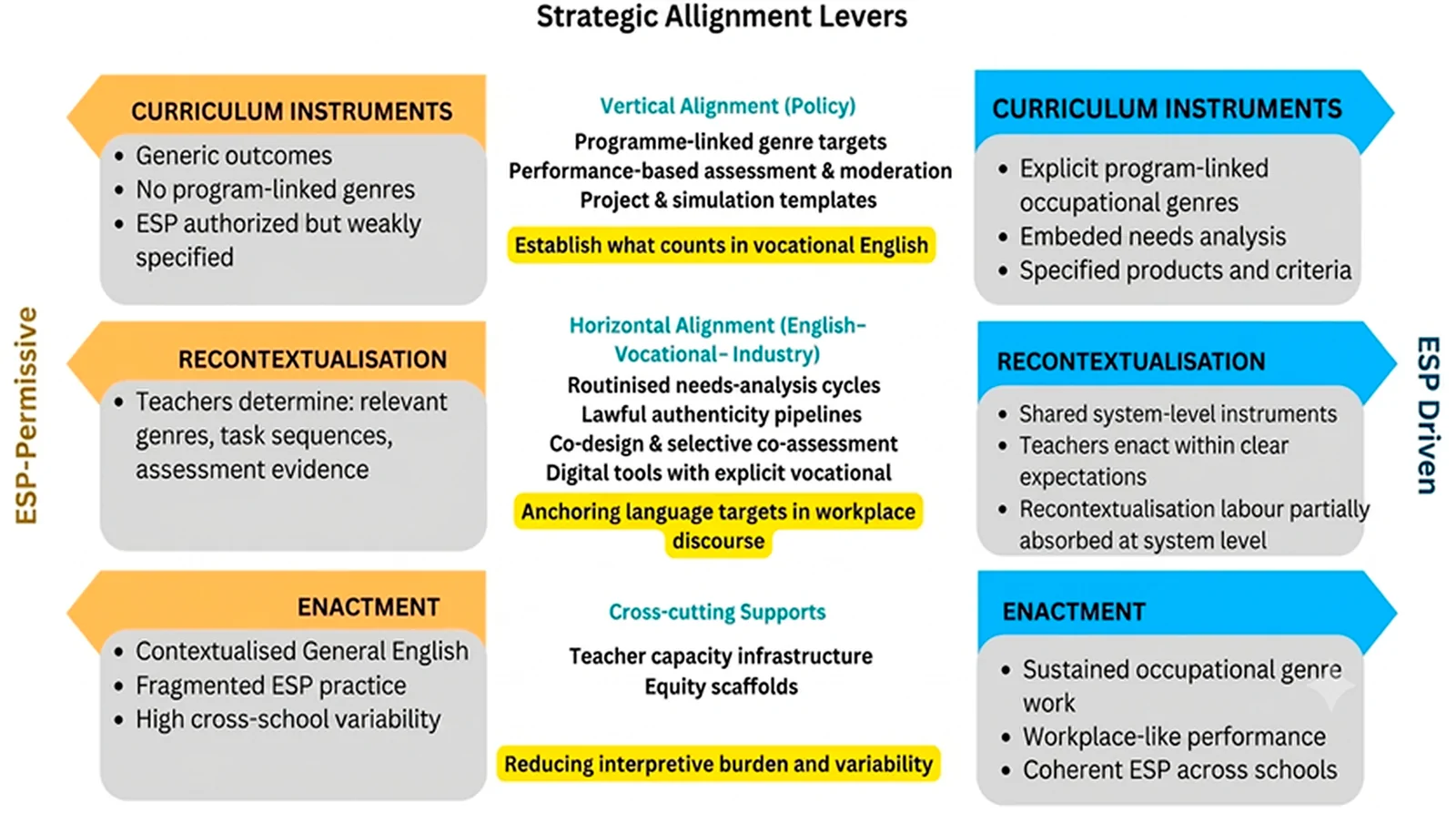
Strategic alignment framework for shifting from an ESP-permissive to an ESP-driven curriculum architecture.

Figure 2 synthesizes how the identified alignment levers function collectively to move the system from an ESP-permissive to an ESP-driven curriculum architecture.

On the left side of the figure, the current ESP-permissive condition is characterised by policy and curriculum instruments that authorise contextualisation but remain weakly specified. In this configuration, critical enactment work such as determining priority occupational genres, sequencing tasks toward workplace performance, and defining valid assessment evidence is displaced to schools and individual teachers. As documented in the findings, this distribution of recontextualisation labour contributes to fragmented ESP practice, frequent defaulting to General English, and substantial variability across schools.

The central panel of Figure 2 presents the strategic alignment framework, which translates these diagnostic findings into concrete design levers. These levers operate along two interrelated dimensions: vertical alignment and horizontal alignment. Vertical alignment concerns coherence from policy and curriculum frameworks through learning modules, pedagogy, and assessment. In this dimension, the framework emphasises the specification of programme-linked priority occupational genres, explicit mapping from learning outcomes to assessable products and performance conditions, the adoption of moderated performance-based assessment, and the use of project and simulation templates as default instructional vehicles. Together, these instruments stabilise what counts as vocational English performance and absorb part of the interpretive work currently borne by teachers.

Horizontal alignment concerns coordination across English, vocational subjects, and industry. Here, the framework foregrounds routinised needs-analysis cycles, lawful infrastructures for accessing authentic workplace artefacts, formalised co-design and selective co-assessment routines, and the explicit vocational framing of digital tools. These mechanisms anchor language targets in authentic workplace discourse and institutionalise collaboration beyond individual initiative. Cross-cutting supports, including ESP-focused teacher capacity development and equity scaffolds such as shared task banks and low-resource simulation options, further reduce variability and mitigate inequities in implementation capacity across schools.

The right side of Figure 2 represents the target condition: an ESP-driven curriculum architecture. In this configuration, ESP is stabilised through explicit curriculum instruments, routinised feedback loops with industry, and shared assessment expectations. Recontextualisation labour is no longer concentrated at the classroom level but is partially absorbed at the system level, enabling teachers to enact vocational ESP within clearer, shared parameters. The expected outcome is more coherent occupational genre work, the production of workplace-like performance evidence, and greater comparability of vocational English learning opportunities across contexts.

Table 5 elaborates the strategic framework by specifying each design lever, its operational indicators, the empirical rationale drawn from the findings, and corresponding implementation implications **.** Read together, Figure 2 and Table 5 demonstrate that progress toward ESP-driven implementation depends not on isolated innovations, but on the coordinated activation of vertical and horizontal alignment mechanisms that collectively reshape how vocational intent is translated into classroom practice.

**Table 5.** ESP-Driven design principles: Vertical and horizontal alignment levers.

Design lever	What to specify (indicator)	Evidence from findings (why this lever is needed)	Implementation implication
1.Program-linked genre targets	A program-linked set of priority workplace genres and communicative events (e.g., email sequences, memos/minutes, invoices/quotations, service encounters)	Documents do not specify vocational genres; stakeholders request concrete workplace genres	Provide genre lists with brief descriptors to anchor *ATP, Modul Ajar, * and assessment tasks
2. Outcomes-to-products mapping	Each unit specifies target product(s), performance conditions, and criteria aligned to outcomes	Outcomes are perceived as broad; teachers report reworking *CP* into programme-specific *ATP*	Use unit templates that link outcomes to products, performance conditions, and criteria, and specify the expected evidence
3.Routinised needs analysis cycle	A scheduled needs analysis cycle combining internship feedback, employer consultation, and genre sampling	Needs analysis is informal or absent	Standardize needs analysis instruments and reporting, and use results to revise *ATP* and tasks
4.Lawful authenticity pipeline	MoU templates and anonymisation protocols enabling lawful access to workplace artefacts	Authenticity is valued but legally constrained; teachers rely on ad hoc searching	Establish secure channels and repositories for anonymised artefacts, task briefs, and exemplars
5. Performance-based assessment regime	Workplace-like performance tasks supported by shared rubrics and moderation routines	Workplace performance evidence is weak; assessment is often generic	Introduce shared rubrics and moderation to strengthen comparability across schools
6. Projects and simulations as default vehicles	Project and simulation templates specifying sequences, roles, artefacts, and assessment criteria	Projects are reduced due to feasibility constraints	Provide ready-to-use briefs and sequencing exemplars aligned to programme-linked genres
7. Horizontal coordination routines	Scheduled co-design and selective co-assessment among English teachers, vocational teachers, and industry partners	Cross-subject linkage is weak; stakeholders propose co-design and co-assessment	Institutionalise collaboration windows and a semester coordination calendar
8. Teacher capacity infrastructure	ESP-focused professional development in genre pedagogy, task design, and assessment moderation	Teachers shoulder the recontextualisation workload	Build professional development pathways and mentoring focused on vocational genre teaching and performance assessment
9.Equity scaffolds	Shared task banks, exemplars, and low-resource simulation options	Variability and inequity risk across schools	Provide scalable packages and minimum-quality templates to support under-resourced schools
10. Digital tools with explicit vocational purpose	Digital activities explicitly linked to workplace communication functions and assessed accordingly	Digitalization often lacks vocational purpose	Require vocational purpose statements in Modul Ajar and include them in assessment criteria

Rather than a fixed national blueprint, these levers are offered as a tentative and adaptable framework to guide policy refinement and future research. The framework highlights that progress toward ESP-driven implementation depends on tightening vertical alignment from policy to assessment while institutionalising horizontal coordination among English, vocational subjects, and industry.

## 6. Implications

### 6.1 Theoretical implications

**6.1.1 Curriculum architecture as an analytic lens for ESP policy**

The ESP-permissive/ESP-driven distinction advances ESP and language policy theorising by reframing “ESP orientation” as an enactment property rather than a mere policy aspiration. In *ESP-permissive* curricula, ESP is formally authorized but weakly instrumented: the policy permits contextualization yet leaves critical enactment work—such as specifying priority occupational genres, operationalizing needs analysis routines, and defining performance evidence—to local interpretation. In *ESP-driven* curricula, ESP is stabilized through explicit instruments (e.g., programme-linked genre targets, embedded needs-analysis cycles, product/performance specifications, and shared assessment evidence), which reduces ambiguity and enables more consistent enactment across sites.

Crucially, this framework makes visible a key mechanism in policy enactment: the distribution of recontextualisation labour across system levels. When curriculum instruments remain generic, the burden of translating policy into teachable content and assessable performance is displaced to schools and teachers, increasing variation in what counts as “ESP” in practice. Conversely, when instruments are explicit and routinized, a portion of this interpretive work is absorbed at the curriculum and governance level, creating clearer conditions for coherent ESP implementation. The distinction therefore offers a transferable typology for diagnosing why vocational intent can persist alongside General English reliance, shifting ESP policy analysis from stated goals to the mechanisms of enactment that enable or constrain specialization.

**6.1.2 Instrument specificity and recontextualisation dynamics**

The findings support the proposition that where curriculum instruments remain generic, the work of recontextualising policy is displaced to schools and teachers, contributing to uneven enactment. In such settings, teachers must independently determine (i) which workplace genres matter, (ii) how to sequence tasks toward occupational performance, and (iii) what counts as valid evidence of competence. This provides a theoretically grounded explanation for why vocational intent can coexist with continued reliance on General English: without specific instruments, “ESP” tends to be interpreted as contextual examples within GE rather than as a structured genre-and-task progression anchored in vocational discourse.

**6.1.3 Twin alignments as coherence conditions for vocational english**

The study conceptualizes coherence in vocational ESP as requiring twin alignments both vertical and horizontal thereby extending conventional “alignment” arguments beyond internal document consistency. Vertical alignment concerns coherence across policy, curriculum frameworks, modules, pedagogy, and assessment so that stated outcomes translate into specified products and performance conditions. Horizontal alignment concerns coordination across English, vocational subjects, and industry discourse so that language targets reflect authentic workplace practices and are sustained by institutional partnership routines. Together, these alignments function as coherence conditions that reduce fragmentation and strengthen the plausibility of vocational ESP as a system-wide practice rather than a teacher-dependent initiative.

### 6.2 Practical and policy implications: operationalizing twin alignments

The recommendations below are offered as design levers for strengthening coherence in vocational ESP implementation. They are not proposed as a definitive national blueprint, but as mechanisms that can be adapted, piloted, and refined across contexts.

**6.2.1 Vertical alignment (Policy, Curriculum Frameworks, Teaching Modules, And Assessment)**

**6.2.1.1 Specify programme-Linked priority occupational genres.**

Strengthen curriculum guidance by identifying a small set of priority occupational genres and communicative events per vocational cluster (e.g., email sequences, memos/minutes, invoices/quotations, SOP summaries, service encounters), accompanied by brief descriptors of purpose, audience, and core conventions.

**6.2.1.2 Require explicit mapping from outcomes to products and performance conditions.**

In syllabus and lesson plans, require each unit to specify target genre(s), intended product(s), and basic performance conditions (e.g., tools, time constraints, interactional format). This makes expectations transparent and reduces interpretive ambiguity.

**6.2.1.3 Adopt moderated performance-Based assessment with shared rubrics.**

Align assessment with workplace communication by introducing performance tasks that mirror occupational sequences (e.g., document set to email response; client call to minutes and follow-up message) and evaluating them using shared rubrics (clarity, completeness, appropriacy of register/tone, accuracy, and formatting). Moderation (sampling and calibration) can support comparability and reduce local arbitrariness.

**6.2.2 Horizontal alignment (English, Vocational Subjects, Industry)**

**6.2.2.1 Institutionalize routine Needs-Analysis Cycles.**

Establish scheduled needs-analysis cycles (e.g., semester/annual) that combine internship feedback, employer consultation, and genre sampling/document review. This provides an auditable basis for revising genre priorities and communicative scenarios in line with workplace practices.

**6.2.2.2 Standardize partnership infrastructure for lawful access to workplace artefacts.**

Develop standard MoU templates and anonymization protocols that enable industry partners to share workplace artefacts (emails, forms, SOP excerpts, logs) in legally and ethically acceptable forms, supporting systematic incorporation of authentic discourse.

**6.2.2.3 Formalize co-design and selective co-assessment routines.**

Create scheduled collaboration windows among English teachers, vocational subject teachers, and (where feasible) industry partners for project brief design, feedback cycles, and selective co-assessment of key performance tasks. This strengthens the linkage between language outcomes and vocational competence expectations. Implementation feasibility is strengthened by targeted capacity-building in genre-based pedagogy, task design, and assessment moderation, enabling system expectations to be enacted with reduced interpretive burden at school level.

## Data Availability

The datasets generated and analysed during the current study are not publicly available due to ethical, privacy, and institutional confidentiality considerations. The data include semi-structured interview transcripts, school-level curriculum documents, and field-related materials that may contain information capable of identifying participants, schools, or partner institutions. Some industry-related information may also contain context-specific or institutionally sensitive details. Therefore, the full raw data cannot be deposited in a public repository. De-identified excerpts relevant to the analysis are included in the article to support transparency in the presentation of findings. Additional access to de-identified supporting data may be requested from the corresponding author, Prof. Urip Sulistiyo, at urip.sulistiyo@unja.ac.id, or from Prof. Amirul Mukminin, as a member of the research team and data access contact, at amirul.mukminin@unja.ac.id. Requests should include a brief explanation of the intended academic use of the data and will be considered subject to confidentiality requirements, institutional permission, and reasonable academic use. Extended data materials used in the study, including the research permit letter, blank/example informed consent form, interview guides, document analysis guideline, and anonymised supporting materials, have been deposited in Zenodo and are available at: https://doi.org/10.5281/zenodo.20680968 [ Putimasurai, Sulistiyo, U., & Mukminin, A. (2026)] Creative Commons Attribution 4.0 International. The full interview transcripts are not publicly available because public release could compromise participant, school, or partner-institution confidentiality.

## References

[ref1] Albesher KB : Inclusion of English for specific purposes in high school tracks: A Saudi context. *International Journal of English Language and Literature Studies.* 2023;12(4):342–357. 10.55493/5019.v12i4.4904

[ref2] Anthony L : *Introducing English for Specific Purposes.* Paltridge B Starfield S , editors. Routledge;2018. 10.4324/9781351031189

[ref3] Arnó-Macià E Aguilar-Pérez M Tatzl D : Engineering students’ perceptions of the role of ESP courses in internationalized universities. *Engl. Specif. Purp.* 2020;58:58–74. 10.1016/j.esp.2019.12.001

[ref34] Aminah M Suseno M Setiadi S : Exploring the implementation process of an ESP program for engineering students: A CIPP-based evaluation. *IDEAS: Journal on English Language Teaching and Learning, Linguistics and Literature.* 2025;13(2):4866–4875.

[ref35] Asrifan A Barros Cardoso LMO de : The role of multiliteracies in enhancing the ESP curriculum at vocational high schools. *Jurnal Pendidikan Progresif.* 2025;15(3):1953–1969. 10.23960/jpp.v15i3.pp1953-1969

[ref4] Badan Standar Kurikulum dan Asesmen Pendidikan: Kajian akademik Kurikulum Merdeka. 2024.

[ref5] Basturkmen H : ESP research directions: Enduring and emerging lines of inquiry. *Language Teaching Research Quarterly.* 2021;23:5–11. 10.32038/ltrq.2021.23.02

[ref6] Basturkmen H : Current trends in ESP research in the Asia Pacific region. *World Englishes.* 2022;41(4):512–522. 10.1111/weng.12601

[ref7] Beck L : Curriculum form and professional practice. *Discourse: Studies in the Cultural Politics of Education.* 2025;47:274–288. 10.1080/01596306.2025.2551362

[ref36] Bernstein B : Pedagogy, symbolic control and identity: Theory, research, critique. *Revised ed. Rowman & Littlefield;* 2000.

[ref8] Bhatia VK : *Analysing genre: Language use in professional settings.* Routledge;1993.

[ref37] Bowen GA ;2000: Document analysis as a qualitative research method Qualitative Research Journal;2009.9(2):27–40. 10.3316/QRJ0902027

[ref38] Creswell JW : *Qualitative inquiry and research design: Choosing among five approaches.* 3rded: SAGE Publications;2013.

[ref9] Dickens S : Intentional, tacit, contingent: Knowledge recontextualisation in the “official” curriculum. *J. Curric. Stud.* 2021;53:692–710. 10.1080/00220272.2021.1904007

[ref10] Dou AQ Chan SH Win MT : Changing visions in ESP development and teaching: Past, present, and future vistas. *Front. Psychol.* 2023;14:1140659. 10.3389/fpsyg.2023.1140659 37138994 PMC10150772

[ref11] Dudley-Evans T St. John MJ : *Developments in English for Specific Purposes: A multi-disciplinary approach.* Cambridge University Press;1998.

[ref12] Hordern J : Recontextualisation and the teaching of subjects. *The Curriculum Journal.* 2021;32:592–606. 10.1002/curj.110

[ref13] Hutchinson T Waters A : *English for Specific Purposes: A learning-centred approach.* Cambridge University Press;1987. 10.1017/CBO9780511733031

[ref14] Hyland K : *English for Academic Purposes: An advanced resource book.* Routledge;2006. 10.4324/9780203006603

[ref39] Hyland K Jiang FK : A bibliometric study of EAP research: Who is doing what, where and when? *Journal of English for Academic Purposes.* 2021;49:100929. 10.1016/j.jeap.2020.100929

[ref15] Hyland K : English for specific purposes: What is it and where is it taking us?. *ESP Today.* 2022;10(2):202–220. 10.18485/esptoday.2022.10.2.1

[ref16] Jiang AL Zhang LJ May S : Understanding novice teachers’ perceived challenges and needs as a prerequisite for English curriculum innovation. *Lang. Cult. Curric.* 2020;33(1):15–31. 10.1080/07908318.2018.1518452

[ref17] Liu Y Hu G : Mapping the field of English for specific purposes (1980–2018): A co-citation analysis. *Engl. Specif. Purp.* 2021;61:97–116. 10.1016/j.esp.2020.10.003

[ref40] Lincoln YS Guba EG : *Naturalistic inquiry.* vol.9: SAGE Publications;1985.

[ref49] Luzón MJ Pérez-Llantada C : Digital genres in academic knowledge production and communication: Perspectives and practices Multilingual Matters;2022. 10.21832/9781788924726

[ref41] Merriam SB Tisdell EJ : *Qualitative research: A guide to design and implementation.* 4thed: Jossey-Bass/Wiley;2015.

[ref18] Misbah Z Gulikers J Maulana R : Evaluating competence-based vocational education in Indonesia. *J. Vocat. Educ. Train.* 2020;72:488–515. 10.1080/13636820.2019.1635634

[ref19] Mukminin A Habibi A Prasojo LD : Curriculum reform in Indonesia: Moving from an exclusive to inclusive curriculum. *Center for Educational Policy Studies Journal.* 2019;9(2):53–72. 10.25656/01:17442

[ref42] Nurisma RA : *Enhancing students’ reading comprehension and attitudes in ESP course through literature circles with self-regulated learning.* : Doctoral thesis, Universitas Negeri Malang;2025.

[ref43] Patton MQ : *Qualitative research & evaluation methods: Integrating theory and practice.* 4thed: SAGE Publications;2015.

[ref20] Pambudi HA Kaliaskarova A : The foundation of English learning to equip vocational students in higher education to face industrial era 4.0. *Jurnal Pendidikan Teknologi dan Kejuruan.* 2023;29(2):230–239. 10.21831/jptk.v29i2.66994

[ref21] Piedra Moreno DP Meaney T Herheim R : Processes of recontextualizing a curriculum reform into local meaning-making. *Instr. Sci.* 2025;28:1441–1465. 10.1007/s10857-025-09686-2

[ref22] Poedjiastutie D Oliver R : English learning needs of ESP learners: Exploring stakeholder perceptions at an Indonesian university. *TEFLIN Journal.* 2017;28(1):1–21. 10.15639/teflinjournal.v28i1/1-21

[ref44] Pratama R Mukminatien N Fatmawati , Suhono: Developing assessment instruments for procedural writing of managing flight emergency and urgency situations: Examining Indonesian aviation English instructors’ perceptions. IDEAS: Journal on English Language Teaching and Learning, Linguistics and Literature. 2025;13(2):5096–5117. 10.24256/ideas.v13i2.7775

[ref23] Purwanti AR : Revisiting English for specific purposes (ESP) in Indonesian vocational high school (VHS): A current situation in curriculum 2013. *ETERNAL: English Teaching Journal.* 2018;9(2):97–108. 10.26877/eternal.v9i2.2984

[ref33] Putimasurai Sulistiyo U Mukminin A : Extended Data for: From ESP-Permissive to ESP-Driven: Recontextualizing English in Indonesia's Vocational Curriculum.[Data set]. *Zenodo.* 2026. 10.5281/zenodo.20680968

[ref45] Rachmawati DL : Optimizing ESP learners’ English vocabulary proficiency through mobile-assisted formative assessment. *International Journal of Pedagogical Language, Literature, and Cultural Studies.* 2025;2(3):145–153. 10.63011/ip.v2i3.40

[ref24] Richards JC : Curriculum approach in language teaching. *RELC J.* 2013;44(1):5–33. 10.1177/0033688212473293

[ref25] Roshid MM Kankaanranta A : English communication skills in international business: Industry expectations versus university preparation. *Bus. Prof. Commun. Q.* 2023;88:100–125. 10.1177/23294906231184814

[ref26] Sari LI Rukmini D Faridi A : English for specific purposes curriculum evaluation from the social semiotic perspective. *International Journal of Evaluation and Research in Education.* 2024;13(4):2719–2730. 10.11591/ijere.v13i4.28014

[ref27] Syahida WQ Siminto S : A review of English for Vocational High School in Kurikulum Merdeka. *Atmosfer: Jurnal Pendidikan, Bahasa, Sastra, Seni, Budaya, dan Sosial Humaniora.* 2023;1(3):127–139. 10.59024/atmosfer.v1i3.220

[ref46] Tracy SJ : *Qualitative research methods: Collecting evidence, crafting analysis, communicating impact.* 2nded: Wiley-Blackwell;2020.

[ref28] UNESCO: *Unleashing the potential: Transforming technical and vocational education and training.* UNESCO Publishing;2015. 10.54675/HXES1603

[ref29] UNESCO: *UNESCO strategy for TVET (2022–2029): Transforming technical and vocational education and training for successful and just transitions.* UNESCO;2022. Reference Source

[ref30] Widodo HP : Language policy in practice: Reframing the English language curriculum in the Indonesian secondary education sector. *English Language Education Policy in Asia.* Springer Nature;2016; Vol.11: pp.127–151. 10.1007/978-3-319-22464-0_6

[ref48] Yin RK : *Case study research and applications: Design and methods.* 6thed: SAGE Publications;2018.

[ref47] Zhang H Jin SJ Du SZ : Developing a curriculum model of English teaching for master’s degree nursing education in a Chinese medicine university. *International Journal of Nursing Sciences.* 2020;7(1):99–104. 10.1016/j.ijnss.2019.12.001 32099866 PMC7031153

[ref32] Zhao L Zhao B Li C : Alignment analysis of teaching–learning–assessment within classroom project-based learning to curriculum standards. *Humanities and Social Sciences Communications.* 2023;5. 10.1186/s43031-023-00078-1

