# Supplementary material for: From ESP-Permissive to ESP-Driven: Recontextualizing English in Indonesia’s Vocational Curriculum
Source: F1000Res. 2026 Jul 27;15:1232. [Version 1] doi: 10.12688/f1000research.182978.1 (PMC13560047; doi:10.12688/f1000research.182978.1)
Supplement: Peer review history

# Reviewer report: Reviewer response for version 1

**DOI:** 10.5256/f1000research.201971.r506396

**Published:** 2026-08-26

**Authors:** Wiwiet Eva Savitri

**Affiliations:** 1 Universitas Negeri Surabaya, West Surabaya, Indonesia

The research paper entitled "From ESP-Permissive to ESP-Driven: Recontextualizing English in Indonesia's Vocational Curriculum" addresses a highly relevant yet relatively underexplored issue in vocational English education in Indonesia. The study demonstrates strong novelty, as research examining ESP from a curricular perspective, particularly this specific transition, remains limited. Its focus moves beyond the common discussion of ESP materials and classroom practices to examine how vocational needs shape curriculum orientation. The topic is therefore both theoretically and practically significant. Supported with relevant references and adopting a rigorous qualitative multiple-case study approach to explore the positioning and implementation of English for Specific Purposes (ESP) in Indonesian vocational high schools, this study provides a significant contribution to the body of knowledge and insight for vocational school policy makers.

**Strengths**

- **Strong novelty**: The study addresses a relatively underexplored area by examining the transition from an ESP-permissive to an ESP-driven curriculum in Indonesian vocational high schools.
- **Practical and policy contribution**: The proposed framework has the potential to inform curriculum policymakers and developers in implementing a more ESP-driven English curriculum in vocational high schools, particularly in aligning English learning with students' vocational and workplace needs.
- **Methodological robustness**: The qualitative case study approach is well suited to investigating how ESP is positioned and implemented in vocational high schools.
- **Data triangulation**: The use of multiple data sources strengthens the credibility and trustworthiness of the findings.
- **Research transparency**: The clear description of purposive sampling, semi-structured interviews, and thematic analysis enhances the methodological transparency of the study.

**Weaknesses**

- **Limited geographical coverage**: The participants were drawn from only one city, which may not fully represent the diverse educational and vocational contexts across Indonesia. Given Indonesia's vast geographical and contextual diversity, the findings should therefore be interpreted with caution in terms of broader applicability.
- **Limited sample**: The study involves a relatively small number of participants, with only 21 stakeholders, which may limit the breadth of perspectives captured.
- **Insufficient linkage to curriculum documents**: The study also needs to clarify which specific statements, provisions, or components within the curriculum documents serve as the basis for addressing the research questions. Establishing this connection would strengthen the interpretation of the findings and demonstrate more clearly how the conclusions are grounded in the curriculum itself.
- **Limited attention to final assessment**: The study does not sufficiently address how English is assessed in the final school examination. Examining whether the assessment reflects students' vocational and workplace needs or remains oriented toward General English would provide a more comprehensive picture of the implementation of an ESP-driven curriculum. The relevant curriculum documents should also be examined to determine how the final assessment is formally positioned.

**Recommendation**

 This manuscript is recommended for minor revision. It makes a valuable contribution to ESP in vocational English curriculum development in Indonesia, particularly through its novel focus on the transition from an ESP-permissive to an ESP-driven curriculum. The minor revisions should include a clear statement of which English curriculum is being examined, detailed quotations or references to the specifics in the curriculum documents that serve as the basis for addressing the research questions, and a clearer explanation of the scope of generalizability given the limited geographical coverage and sample size. Addressing these points would further strengthen the manuscript's clarity, rigor, and contribution.

Is the work clearly and accurately presented and does it cite the current literature?

Yes

If applicable, is the statistical analysis and its interpretation appropriate?

Not applicable

Are all the source data underlying the results available to ensure full reproducibility?

Yes

Is the study design appropriate and is the work technically sound?

Yes

Are the conclusions drawn adequately supported by the results?

Yes

Are sufficient details of methods and analysis provided to allow replication by others?

Yes

Reviewer Expertise:

Material Development, ESP, Teaching and Learning Media, AI in ELT, Listening & Viewing, Extensive Reading

I confirm that I have read this submission and believe that I have an appropriate level of expertise to confirm that it is of an acceptable scientific standard.

# Reviewer report: Reviewer response for version 1

**DOI:** 10.5256/f1000research.201971.r506398

**Published:** 2026-08-10

**Authors:** Krishna Chaitanya Edunuru, Edunuru Krishna Chaitanya

**Affiliations:** 1 Central University of Kashmir, Ganderbal, Jammu and Kashmir, India; 2 English, Central University of Kashmir, Ganderbal, Jammu and Kashmir, India

The research paper titled *“From ESP-Permissive to ESP-Driven: Recontextualizing English in Indonesia’s Vocational Curriculum”* presents a clear and precise study supported by extensive references to current literature. The work demonstrates methodological rigor, with a qualitative multiple case study design effectively employed to examine how English for Specific Purposes (ESP) is positioned and enacted within Indonesia’s vocational high schools (SMK).

**Strengths**

- **Clarity of writing**: The paper is well-written, precise, and supported by extensive references to current literature.
- **Methodological rigor**: The qualitative multiple case study design is appropriate for examining ESP positioning in vocational high schools.
- **Triangulation**: Use of multiple data sources enhances validity, reliability, and objectivity.
- **Transparency**: Clear description of purposive sampling, semi-structured interviews, and thematic analysis provides replicability.
- **Contribution**: The proposed framework offers practical guidance for curriculum refinement and future research.

**Weaknesses**

- **Sample size**: The study is limited by the relatively small number of participants (21 stakeholders).
- **Duration**: Short study period restricts the depth and generalizability of findings.

**Recommendation** The paper makes a valuable contribution to ESP scholarship and vocational curriculum development in Indonesia. While the limitations of sample size and duration should be acknowledged, they do not undermine the overall validity of the findings. The manuscript is suitable for indexing with minor revisions addressing the scope of generalizability.

Is the work clearly and accurately presented and does it cite the current literature?

Yes

If applicable, is the statistical analysis and its interpretation appropriate?

Not applicable

Are all the source data underlying the results available to ensure full reproducibility?

Yes

Is the study design appropriate and is the work technically sound?

Yes

Are the conclusions drawn adequately supported by the results?

Yes

Are sufficient details of methods and analysis provided to allow replication by others?

Yes

Reviewer Expertise:

English Language Teaching, English for Specific Purposes. Technology-Integrated Language Teaching, Teachers' Professional Development and Flipped Classroom Pedagogy

We confirm that we have read this submission and believe that we have an appropriate level of expertise to confirm that it is of an acceptable scientific standard.
